# Incidental detection of PDAC via ^18^F-PSMA PET/CT in a patient with recurrent prostate cancer. A case report

**DOI:** 10.3389/fnume.2026.1767321

**Published:** 2026-03-10

**Authors:** Giordano Savelli, Mattia Bonacina, Alberto Soffientini, Elvira Archiati, Claudio Bnà, Alberto Zaniboni

**Affiliations:** 1 Nuclear Medicine “Alessandra Bono”, Fondazione Poliambulanza Istituto Ospedaliero, Brescia, Italy; 2 Radiology, Fondazione Poliambulanza Istituto Ospedaliero, Brescia, Italy; 3 Medical Oncology, Fondazione Poliambulanza Istituto Ospedaliero, Brescia, Italy

**Keywords:** pancreatic adenocarcinoma, positron emission tomography, prostate ductal adenocarcinoma, prostate specific membrane antigen, theranostic

## Abstract

**Introduction:**

Prostate specific membrane antigen (PSMA) is a type II transmembrane protein overexpressed in the neovasculature of some pancreatic ductal adenocarcinoma (PDAC). PET/CT can detect this expression and has now become an essential tool in this context. However, this antigen can also be expressed by other neoplasms. While this may create diagnostic uncertainty, it could also open therapeutic opportunities.

**Clinical case:**

Here we describe a case in which a PET/CT performed to restage a patient with prostate adenocarcinoma experiencing biochemical recurrence revealed the coexistence of a PDAC.

**Literature review:**

A literature review aimed to summarize the bibliographic evidence on the use of this technique in this setting, which is relatively uncommon.

**Conclusion:**

Prostate-specific membrane antigen can be overexpressed in PDACs. This finding may offer potential for theranostic applications

## Introduction

### Patient history

A patient with high-risk prostate cancer [GS 5 + 4, cT3bN0M0, initial Prostate Specific Antigen (PSA) 11.2 ng/mL], previously treated with radiation therapy (73.5 Gy/30 fractions to the prostate bed and 54 Gy/30 fractions to seminal vesicles) and LHRH analog therapy, came to our attention for suspected biochemical recurrence.

### Laboratory findings

A few weeks after radiotherapy, PSA dropped to 0.2 ng/mL, then began to rise again to 0.5 ng/mL (six months later), 0.3 ng/mL (one year), 1.5 ng/mL (eighteen months), and finally 2.9 ng/mL (shortly before the PET/CT).

### Imaging findings

PET/CT (Siemens Biograph mCT 40 PET/CT scanner) examination was carried out nearly 90 min after the i.v administration of 335 MBq of ^18^F-DCFPyL (Pylclari CURIUM France) (4.5 MBq/kg). Imaging consisted of a single static whole-body acquisition, covering six bed positions with an acquisition time of 2.5 min per bed. A low-dose CT (100–120 kV, modulated mAs, 2–3 mm slices) was acquired for attenuation correction and anatomical localization. PET/CT data were reconstructed using an ordered subset expectation maximization algorithm, on a 256 × 256 matrix.

**Figure 1:**
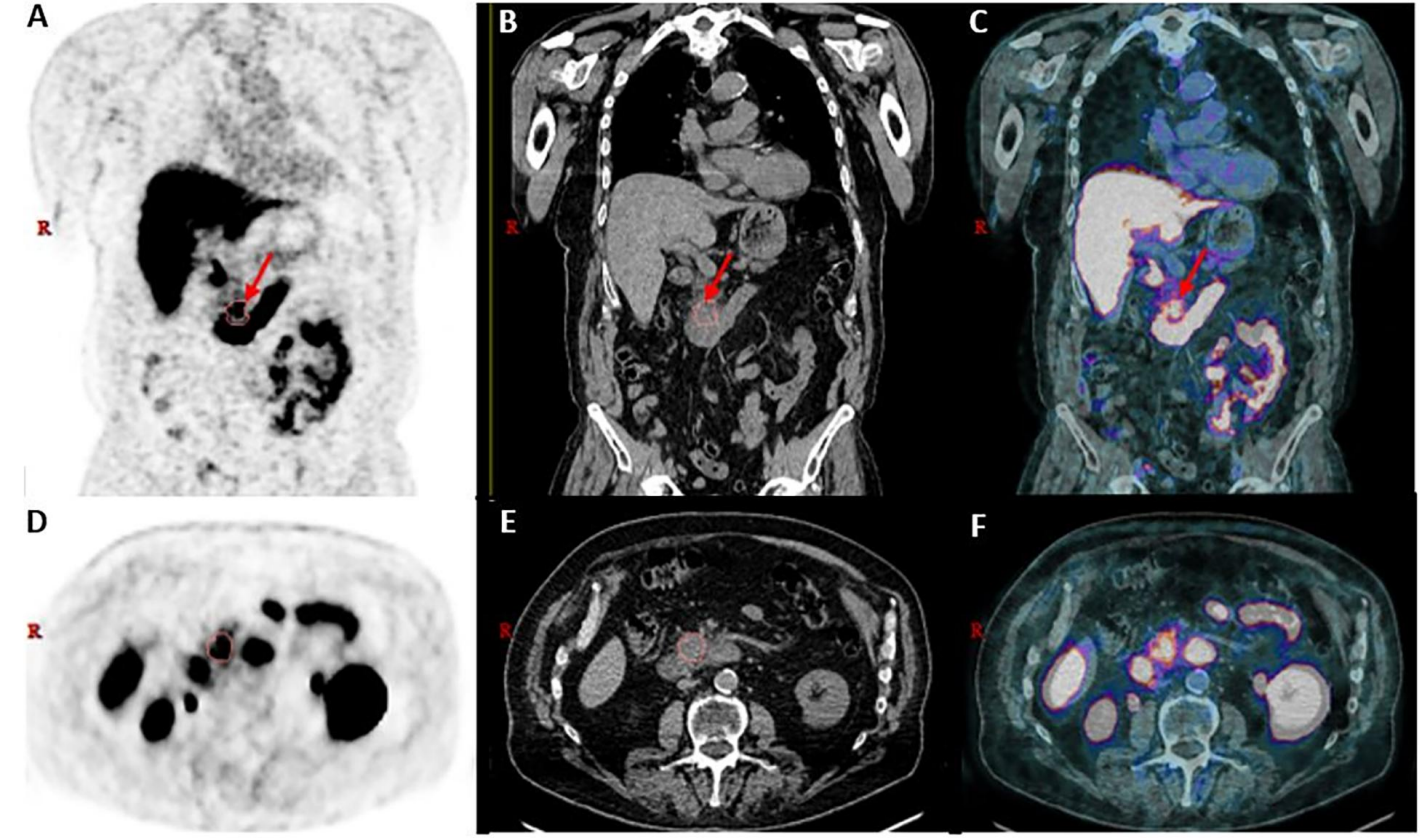
18F-DCFPyL PET/CT images showing uptake in the pancreatic uncinate process (orange borders/red arrow). **(A–C)** Coronal PET, CT, and fused PET/CT. **(D–F)** Transaxial PET, CT, and fused PET/CT.

The scan confirmed recurrence in the prostate and bone metastases. Incidentally, a large area of moderate-to-intense uptake was noted in the head/uncinate process of the pancreas, raising suspicion for a secondary neoplasm at this site (Figures 1A–C).

Following the PET/CT finding, and while awaiting clarification of the nature of the pancreatic uptake, the patient was treated with leuprolide acetate 11.25 mg i.m. to be continued every 3 months and apalutamide.

### Histopathology

Pancreatic biopsy revealed the presence of scattered atypical cell clusters and fibrous tissue containing a modest glandular population of adenocarcinomatous nature. Core needle biopsy fragments were represented predominantly by fibrino-inflammatory and necrotic material, partly by fibrous stroma within which some atypical epithelial fragments are embedded with dysmorphic nuclei and cytoplasmic vacuolization. Epithelial fragments with lesser cytonuclear atypia were present. The established diagnosis was PDAC.

### Treatment and outcome

An initial assessment with abdominal ultrasound revealed no pancreatic abnormalities. Unfortunately, the follow-up instrumental examinations were delayed by the significant pressure on the Italian national health system, and as a result, the MRI was conducted approximately six weeks after the PET scan. MRI evidenced an ovoid 28 × 21 mm lesion in the uncinate process with irregular margins, hypoenhancement, and mild diffusion restriction, consistent with neoplasm suggestive for a pancreatic neoplasm, presumably PDAC.

Approximately one month after the MRI, the established pathologic diagnosis was PDAC. Following the PDAC pathologic diagnosis, the patient was admitted for cancer cachexia. Shortly after the final diagnosis, due to the rapid decline in physical condition, the patient was admitted to hospice care. The patient passed away approximately two months later.

### Literature review

Observations and studies regarding the presence of PSMA in exocrine pancreatic tumors have been, for the most part, preclinical or based on occasional findings. In 2014, Ren et al. studied the expression of PSMA in cancer tissues, pancreatic intraepithelial neoplasia, and normal pancreatic tissues (1). The expression of PSMA and mRNA was detected by immunohistochemistry and real-time quantitative polymerase chain reaction. The conclusion was that PSMA is involved in the carcinogenesis of pancreatic cancer, and it might serve as a potential therapeutic target for pancreatic cancer. In 2017, Stock et al. analyzed PSMA expression in 81 PDAC tissue samples from 61 patients (2). PSMA was practically absent in tumor tissue and PDAC cell lines (0/7) but could be detected in tumor-associated neovasculature in nearly half of the specimens. Moreover, PSMA-positive neovessels correlated with survival. They concluded that PSMA expression in tumor-associated neovasculature is a common feature and that response to therapy might be based on enhanced intratumoral bioavailability of systemic chemotherapy. The same year, Chan et al. reported the detection of a serous cystadenoma (a benign tumor) during a ^68^Ga-PSMA PET/CT performed for staging of prostate carcinoma (3). The first report of PDAC detection by ^68^Ga-PSMA PET/CT is attributed to Sahbai et al., during restaging of a patient with persistently detectable PSA levels (11 ng/mL) 6 months after radical prostatectomy for Gleason 4 + 4 adenocarcinoma (4). The area of uptake in the pancreas corresponded to a pancreatic soft tissue mass of 32 × 22 mm on contrast-enhanced CT (SUV peak 7.6). The patient underwent Whipple operation followed by adjuvant chemotherapy. However, histological examination did not reveal PSMA expression in tumor cells. Therefore, the researchers concluded that the uptake was due to PSMA presence in the tumor neovasculature. A second report is from Sirtl et al. again involving a PDAC, this time located in the uncinate process of the pancreas (5).

All these articles describe incidental findings of pancreatic neoplasms on PSMA PET/CT imaging.

The first (and so far, only) study aimed at evaluating the use of PET/CT with a PSMA-binding radiopharmaceutical (in this case ^18^F-DCFPyL) was that of Puik et al. in 2025. The study enrolled seventeen patients with clinically resectable PDAC who underwent ^18^F-DCFPyL PET/CT prior to surgery. SUVmax and tumor-to-background ratio (TBR) were calculated. Tracer uptake on PET/CT was correlated with tissue expression of PSMA in surgical specimens. The authors concluded that although ^18^F-DCFPyL PET/CT was able to detect 13 out of 17 PDAC cases (76%), the uptake was generally low and lacked specificity. Moreover, no significant uptake was observed in lymph nodes or distant metastases. Therefore, they concluded, the clinical utility of ^18^F-DCFPyL PET/CT imaging in this setting appears to be limited (6).

## Discussion

In the last ten years, imaging using PSMA-targeted radiopharmaceuticals has emerged as an essential tool in prostate adenocarcinoma management, initially for diagnosis and subsequently for theranostic applications. Biochemically, PSMA functions as a type II transmembrane zinc metallopeptidase with enzymatic capabilities, specifically hydrolyzing poly-*γ*-glutamated folates into folate. Under normal physiological conditions, PSMA expression occurs in astrocytes and Schwann cells within the nervous system, as well as in the prostate, proximal renal tubules, salivary glands, and the duodenal brush border. Despite its nomenclature, PSMA is far from being truly “prostate-specific”. This ‘non-prostate-specific’ nature certainly represents a factor to consider when analyzing its biodistribution (i.e., during the diagnostic phase). However, the fact that PSMA is also overexpressed by other neoplasms also represents a potential opportunity. This consideration is even more relevant for highly aggressive malignancies such as PDAC.

**Figure 2:**
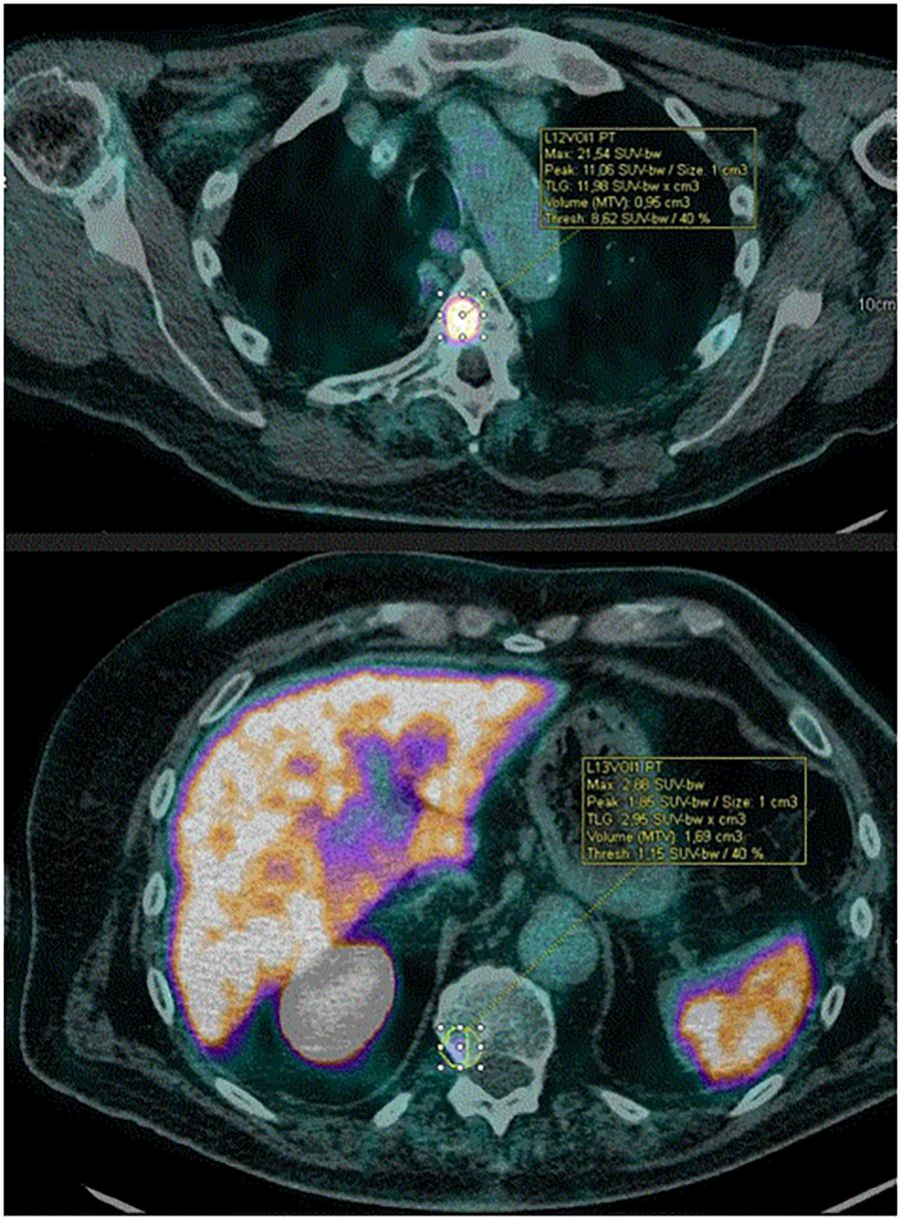
VOIs of bone metastases showing the least avid lesion (lower panel) and the most avid lesion (upper panel). Pancreatic uptake intensity falls between these two extremes.

In this patient's case, in addition to identifying the secondary localizations of the prostate tumor, the ^18^F-DCFPyL PET/CT showed one area of uptake in the pancreatic head region (SUVpeak of 3.48) which is comparable with that of bone metastases of prostatic origin from the same patient (SUVpeak ranging from 1.85 to 11.46, Figure 2). Prostate metastases to the pancreas are extremely rare (7, 8), thus making this finding highly suspicious for primary pancreatic cancer, which was subsequently confirmed by biopsy.

As previously mentioned, prior studies investigating PSMA in PDAC have shown conflicting results, specifically in terms of the presence or absence of the antigen, its potential localization (neoplastic cells, tumor neovasculature), and its expression density. All considerations reached obviously maintain their importance.

Another topic is represented by which of the numerous available tracers, often having quite different chemical structures, has greater affinity for PSMA and whether, this has an effect, from a clinical point of view, in PDAC. The possible different affinities of the various tracers for PSMA have not been thoroughly studied and compared. However, it appears that the differences regarding tracer-PSMA binding, although present, are not significant enough to have clinical implications (9–11).

Of particular importance is the Puik et al. study (6), the first to be structured as a true clinical trial. The final conclusions are far from optimistic, as they do not recognize any real clinical significance to the presence of PSMA in these tumors. In particular, the Authors emphasize as negative aspects both the relative tumor uptake of the radiopharmaceutical and the high duodenal uptake. From a theranostic perspective, in fact, low tumor uptake combined with high uptake in a critical organ would be associated with excessive toxicity and would minimize the therapeutic index. We agree with the Authors’ considerations. However, the study in question employed only a single tomographic time point (one and a half hours after administration). Pharmacokinetic studies demonstrate that PSMA kinetics are best described by an irreversible two-tissue compartment model. The first compartment represents the blood pool where the radiopharmaceutical is injected, while the second represents PSMA-expressing tumor tissue. The model is considered irreversible because once bound, the radiopharmaceutical becomes trapped intracellularly with negligible washout. Consequently, the uptake in PSMA-expressing tumors increases continuously over time. This behavior is not the same in the duodenum in which the uptake is likely attributed to the dietary uptake of folates in this region and thus increased PSMA expression. While tumor uptake increases if the radiopharmaceutical circulates in blood, duodenal uptake remains essentially stable. A PET/CT scan performed at three hours post-injection would likely show different pancreatic tumor-to-duodenum ratios compared to one-hour imaging. This hypothesis, however, requires prospective validation through dedicated imaging studies with serial time points.

PSMA expression in tumor neovasculature has also been described in other malignancies including hepatocellular carcinoma (12–14), adenoid cystic carcinoma (15, 16), non-small cells lung cancer (17), colorectal cancer and its hepatic metastasis (18), esophageal cancer (19), renal cell carcinoma (20). Preclinical (21) and clinical (22–25) preliminary theranostic evaluations with ^177^Lu-PSMA–based approaches have been carried out in some of these PSMA-positives neoplasms with sometimes encouraging results.

Emerging clinical evidence suggests that PSMA expression in non-prostatic malignancies may offer theranostic opportunities worthy of further investigation. This potential is further enhanced by the development of PSMA-targeted radiopharmaceuticals labeled with alpha-particle or Auger electron emitters, which demonstrate significantly higher relative biological effectiveness (RBE) compared to beta-emitters such as ^1^⁷⁷Lu. Should this approach prove applicable to pancreatic neoplasms, rigorous validation through dedicated prospective studies would be essential.

## Conclusion

This case report reports an unusual finding at ^18^F-DCFPyL PET/CT which revealed to be a PDAC at biopsy. Unfortunately, immunohistochemical analysis was not performed to determine PSMA distribution within the specimen (i.e., neoplastic cells vs. tumor-associated neovasculature), which constitutes an important limitation of this report. However, based on existing literature, uptake was most likely localized to the tumor neovasculature.

What lessons emerge from this case report? Firstly, an imaging study performed for one pathology incidentally revealed a second, more aggressive malignancy. Although such incidental detections have been documented in the literature, in this particular case the discovery of the pancreatic neoplasm substantially worsened the patient's overall prognosis. Despite the inherent limitations of a single case report, and considering the existing scientific literature on PSMA theranostic, an important hypothesis emerges: PSMA expression in PDAC neovasculature may represent a potentially valuable theranostic target deserving rigorous investigation in the perspective of a hypothetical antiangiogenic therapeutic approach (26–29). In selected contexts, this antigen may prove valuable in treating several malignancies, including PDAC.

## Data Availability

The raw data supporting the conclusions of this article will be made available by the authors, without undue reservation.
