# Supplementary material for: Incidental detection of PDAC via 18F-PSMA PET/CT in a patient with recurrent prostate cancer. A case report
Source: Front Nucl Med. 2026 Mar 10;6:1767321. doi: 10.3389/fnume.2026.1767321 (PMC13008924; doi:10.3389/fnume.2026.1767321)
Supplement: Supplementary file 1 [file Table1.docx]

| **Time point** | **Event** | **PSA (ng/mL)** |  |
| --- | --- | --- | --- |
| Baseline | Prostate cancer diagnosis (GS 5+4) | 11.2 |  |
| T0 | Radiotherapy + LHRH therapy |  |  |
| T0 + few weeks | PSA nadir | 0.2 |  |
| T0 + 6 months | Biochemical recurrence | 0.5 |  |
| T0 + 12 months | - | 0.3 |  |
| T0 + 18 months | - | 1.5 | Suspected biochemical recurrence |
| T0 + ~24 months | 18F-DCFPyL PET/CT → PDAC detection | 2.9 | Prostate recurrence, bone metastases, and incidental pancreatic uptake detected |
| T0 + ~24 months | Initial pancreatic assessment |  | Abdominal ultrasound: no abnormalities |
| T0 + ~25.5 months | Further pancreatic characterization |  | MRI: 28×21 mm lesion in uncinate process |
| T0 + ~26 months | Pathological diagnosis |  | Pancreatic biopsy: PDAC confirmed |
| T0 + ~26 months | Clinical deterioration |  | Hospital admission for cancer cachexia |
| T0 + ~26 months | End of life care |  | Hospice care admission |
| T0 + ~28 months | Patient death |  |  |
